# Nasogastric tube dislodgement: a problem on our ICU

**DOI:** 10.1186/cc12180

**Published:** 2013-03-19

**Authors:** B Morton, R Hall, T Ridgway, O Al-Rawi

**Affiliations:** 1 Liverpool Heart & Chest, Liverpool, UK

## Introduction

It was noted on our unit that dislodgement of nasogastric tubes occurred commonly. This can lead to an increased risk of aspiration, interruptions in nutritional support, skin breakdown and radiographic exposure [1]. It is recommended that the position of nasogastric tubes should be confirmed by aspiration and pH testing, with radiographic confirmation used only when this is not possible [2].

## Methods

We performed a retrospective review of chest X-ray (CXR) requests for the 3-month period June to August 2012 using the trust radiology information system. The proportion of CXR requests for confirmation of position and patient demographics were measured with an estimation of the financial cost performed.

## Results

There were 541 patients admitted to the critical care area in the study period. In total, 207 out of 2,340 (8.8%) CXRs performed were for confirmation of position. Repeated X-rays were required in some patients (see Table 1); these patients were older and tended to have a longer length of stay. A mobile CXR costs £25 in our trust, if one CXR is accepted per patient with a nasogastric tube; there was an excess of 160 images with a cost of £4,000 in the 3-month period.

**Table 1:** 

CXR for NG	Patients	Total CXR	Total NG CXR	Proportion	Mean days from first to last CXR	Mean age
1	47	47	321	0.15	8.45	67.55
2	16	32	181	0.18	15.94	69.63
3	7	21	87	0.24	17.86	70.00
4	10	40	149	0.27	15.90	69.20
5	3	15	52	0.29	23.33	66.00
6	3	18	61	0.30	40.00	76.67
8	2	16	52	0.31	39.50	80.00
9	2	18	46	0.39	29.50	83.00

## Conclusion

An excess of CXRs were performed for confirmation of nasogastric tube in our patient population. The recommended methods for position confirmation were reinforced amongst medical staff. The high number of repeated imaging for some patients indicates that dislodgement of tubes was also a problem. We propose that nasogastric tubes should be bridled after first dislodgement or at tracheostomy insertion to minimise dislodgement in the future.
